# KIF3C Promotes Proliferation, Migration, and Invasion of Glioma Cells by Activating the PI3K/AKT Pathway and Inducing EMT

**DOI:** 10.1155/2020/6349312

**Published:** 2020-10-23

**Authors:** Yang Gao, Hui Zheng, Liangdong Li, Changshuai Zhou, Xin Chen, Xiaoyan Zhou, Yiqun Cao

**Affiliations:** ^1^Department of Neurosurgery, Fudan University Shanghai Cancer Center, Shanghai 200032, China; ^2^Department of Oncology, Shanghai Medical College, Fudan University, Shanghai 200032, China; ^3^Department of Nuclear Medicine, Shanghai Tenth People's Hospital, Tongji University, Shanghai 200072, China; ^4^Institute of Pathology, Fudan University Shanghai Cancer Center, Shanghai 200032, China

## Abstract

Kinesin superfamily protein 3C (KIF3C), a motor protein of the kinesin superfamily, is expressed in the central nervous system (CNS). Recently, several studies have suggested that KIF3C may act as a potential therapeutic target in solid tumors. However, the exact function and possible mechanism of the motor protein KIF3C in glioma remain unclear. In this study, a variety of tests including CCK-8, migration, invasion, and flow cytometry assays, and western blot were conducted to explore the role of KIF3C in glioma cell lines (U87 and U251). We found that overexpression of KIF3C in glioma cell lines promoted cell proliferation, migration, and invasion and suppressed apoptosis, while silencing of KIF3C reversed these effects. Ectopic KIF3C also increased the expression of N-cadherin, vimentin, snail, and slug to promote the epithelial-mesenchymal transition (EMT). Mechanistically, overexpression of KIF3C increased the levels of phosphatidylinositol 3-kinase (PI3K) and phosphorylated protein kinase B (p-AKT). These responses were reversed by KIF3C downregulation or AKT inhibition. Our results indicate that KIF3C promotes proliferation, migration, and invasion and inhibits apoptosis in glioma cells, possibly by activating the PI3K/AKT pathway *in vitro*. KIF3C might act as a potential biomarker or therapeutic target for further basic research or clinical management of glioma.

## 1. Introduction

Glioblastoma multiforme (GBM) is the most common, aggressive, and malignant glioma [1]. Despite comprehensive treatment including maximal surgical resection followed by adjuvant radiotherapy and chemotherapy, GBM patients still suffer from poor prognosis, and the overall median survival is only 14.6 months [2–4]. Thus, it is extremely urgent to explore novel molecular therapeutic targets to improve the overall survival of glioma patients.

Kinesin superfamily proteins (KIFs) are a conserved class of microtubule-dependent motor proteins, which can convert the chemical energy of ATP hydrolysis into mechanical energy to regulate many types of intracellular transport [5, 6]. KIFs are involved in intracellular organelle/macromolecule transport, cytoskeleton dynamics, and cell division and migration [7, 8]. The KIF3 family includes KIF3A, KIF3B, and KIF3C [9, 10]. KIF3A is a microtubule-directed motor subunit that transports the *β*-catenin-cadherin complex in the subcellular space [11]. Mouse mutants lacking KIF3A resulted in embryonic lethality as well as large accumulation of arrestin, opsin, and membranes in the retinal photoreceptor inner segment [12, 13]. In addition, upregulation of KIF3A promotes cell proliferation and invasion in prostate cancer [14]. KIF3B is essential for vesicle transport and membrane expansion during cell mitosis [15]. KIF3B was overexpressed in human hepatocellular carcinoma tissues, and its downregulation might inhibit hepatocellular carcinoma proliferation [16]. Another research has shown that KIF3A is related to KIF3B or KIF3C, while KIF3B and KIF3C have no interaction with each other [17].

In contrast to KIF3A and KIF3B, KIF3C is expressed in the central nervous system (CNS). Its expression is identified not only in neuronal cells but also in astrocytes. These findings suggest that the multiple transport roles of KIFs are common in neurons and glial cells [18, 19]. Until now, little information is available on KIF3C in tumor diseases and CNS disorders. The previous report indicated that KIF3C was an injury-specific kinesin that organized the microtubule cytoskeleton in the growth cone to regulate axon growth and regeneration after injury [20]. Wang et al. found that KIF3C was overexpressed in breast cancer tissues, and downregulation of KIF3C could suppress tumor growth and metastasis in breast cancer by inhibiting TGF-*β* signaling [21]. The upregulation of KIF3C has been identified during neural differentiation [22]. However, the exact function and possible mechanism of the motor protein KIF3C in glioma remain unclear.

In this study, we conducted an *in vitro* study to investigate the function and mechanism of KIF3C in the process of proliferation, migration, invasion, and apoptosis in glioma cells.

## 2. Materials and Methods

### 2.1. Cell Culture

The U87 and U251 cell lines were purchased from the cell library of the Chinese Academy of Sciences. All cell lines were cultured in Dulbecco's modified Eagle's medium (DMEM) (Gibco BRL, Gaithersburg, MD, USA) supplemented with 10% fetal bovine serum (Gibco), glutamine (2 mM, Gibco), streptomycin (100 *μ*g/ml, Gibco), and penicillin (100 U/ml, Gibco). The cultures were incubated at 37°C in a humidified 5% CO_2_ atmosphere.

### 2.2. Western Blot Analysis

Cellular proteins were extracted by using radioimmunoprecipitation assay (RIPA) buffer containing a protease inhibitor cocktail (BestBio, Shanghai, China). Equal amounts of protein were subjected to sodium dodecyl sulfate polyacrylamide gel electrophoresis (SDS-PAGE) and transferred to polyvinylidene fluoride (PVDF) membranes (Millipore, Billerica, MA, USA). The membranes were blocked with blocking buffer (5% nonfat milk powder in TBS containing 0.1% Tween) for 2 h. Then, the membranes were incubated with various primary antibodies followed by the appropriate horseradish peroxidase- (HRP-) conjugated secondary antibodies. Additionally, enhanced chemiluminescence (ECL) (Millipore, Billerica, MA, USA) was used to identify immunoreactive bands. All experiments were repeated three times. The antibodies used in this study are listed in Tables 1 and 2.

### 2.3. Quantitative Real-Time PCR

Total RNA was extracted with the TRIzol reagent (Thermo Fisher, Shanghai, China) and reverse transcribed using All-in-One qPCR Mix (AT341, China). Subsequently, real-time PCR was performed with SYBR Premix Ex Taq (TaKaRa Biotechnology, Dalian, China) using the Illumina Eco Real-Time PCR System (Thermo Fisher, Shanghai, China). The primer sequences used in this study are as follows:

- GAPDH: F 5′-GGAGCGAGATCCCTCCAAAAT-3′
- GAPDH: R 5′-GGCTGTTGTCATACTTCTCATGG-3′
- KIF3C: F 5′-CCGGGCCTCCTATTTGGAGA-3′
- KIF3C: R 5′-TCCTTGATGTAGACGCCAGTC-3′

### 2.4. Transient Transfection

Human KIF3C cDNA was subcloned from the glioma cell lines U87 and U251 into the lentiviral vector pCDH-CMV-MCS-EF1-Puro (GeneChem, Shanghai, China). The cloned primer sequences are as follows:

- F: 5′-ATGGCCAGTAAGACCAAGGC-3′
- R: 5′-TCAAGCGTAGTCTGGGACGT-3′

KIF3C-shRNAs were purchased from GeneChem and expressed in the vector pGIPZ (GeneChem, Shanghai, China). The target sequences are as follows:

- shRNA-1: 5′-AGGAGATTGCCGAGCAGAA-3′
- shRNA-2: 5′-TCGCTAAACGAAGATATTA-3′

The empty vector was used as the negative control.

### 2.5. Migration Assay

A cell migration assay was examined by using a 24-well unit containing 8 *μ*m (pore size) polycarbonate membrane inserts (BD Biosciences, San Jose, CA). Approximately 2.0 × 10^4^ cells were added to the upper chamber in medium, and 600 *μ*l of culture media containing 20% fetal bovine serum was added to the bottom chamber. Cells were allowed to migrate at 37°C for 24-48 h toward the lower reservoir. Cells in the upper chambers were removed, and cells in the bottom chambers were fixed with paraformaldehyde and stained with crystal violet. The experiments were repeated three times with duplicate samples.

### 2.6. Invasion Assay

A transwell chamber invasion assay (Matrigel-coated membrane, BD Biosciences, San Jose, CA) was used to examine the cell invasion assay. Approximately 2.0 × 10^4^ cells were seeded in serum-free medium into the upper chamber and allowed to invade the lower chamber, which contained culture medium with 20% fetal bovine serum. After 24-48 h, cells invading the matrix were then fixed with paraformaldehyde for 15 minutes and stained with crystal violet for 30 minutes. All experiments were repeated three times.

### 2.7. Cell Proliferation Assay

Cell Counting Kit-8 (CCK-8, Dojindo Molecular Technologies) was used for test cell proliferation assays. U87 (control and transfection of KIF3C-siRNA and KIF3C overexpression vectors) and U251 (control and transfection of KIF3C-siRNA and KIF3C overexpression vectors) cells were seeded in 96-well plates at a density of 2 × 10^4^ cells/well in 100 *μ*l of medium and grown overnight. At the indicated time points, CCK-8 was used to measure cell proliferation indices according to the manufacturer's instructions. Next, a 96-well format plate reader (Tecan Sunrise, Switzerland) was adopted to measure the cell numbers in triplicate by measuring the absorbance at a wavelength of 450 nm (OD450) every 12 h for 3 days. The experiments were repeated three times.

### 2.8. Apoptosis Assay

Cell apoptosis was measured by using the annexin V-FITC/PI apoptosis detection kit (BD Biosciences) according to the manufacturer's protocol. The mixture was then shaken thoroughly in a dark room at room temperature for 15 minutes and supplemented with 10 *μ*l PI. Flow cytometry (Beckman Coulter, Inc.) was utilized to analyze cell apoptosis. All experiments were repeated three times.

### 2.9. Statistical Analysis

Statistical analyses were performed with GraphPad Prism 6 and SPSS software 17.0 (SPSS Inc., Chicago, IL, USA). All data are shown as the mean ± SD. In addition, Student's *t*-test or one-way ANOVA was used to evaluate differences between groups. *P* values < 0.05 were considered statistically significant.

## 3. Results

### 3.1. Expression of KIF3C in Glioma Cell Lines after RNA Interference or Plasmid Transfection

**Figure 1:**
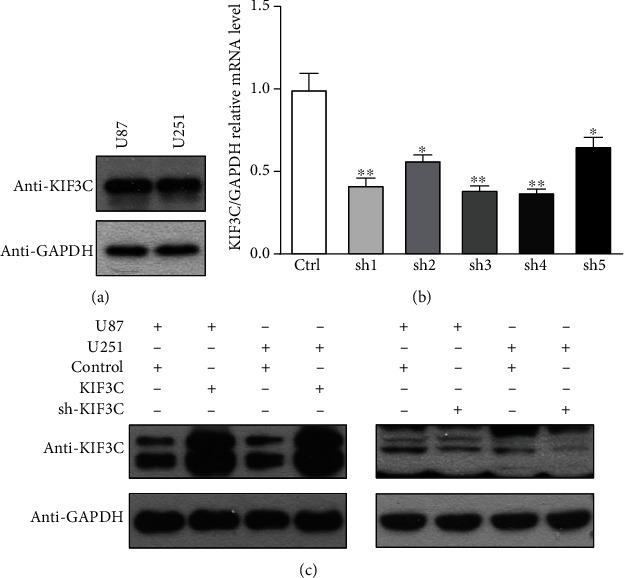
Expression of KIF3C in glioma cell lines. (a) Baseline expression of KIF3C in U87 and U251 cells. (b) Silencing efficiency evaluated by real-time PCR analysis. (c) Silencing and overexpression efficiency of KIF3C evaluated by western blotting analysis in glioma cells. GAPDH was used as the loading control.

The level of KIF3C expression was identified by western blot analysis in U87 and U251 glioma cell lines (Figure 1(a)). To investigate the role of KIF3C *in vitro*, we used an shRNA targeting KIF3C in U87 and U251 cell lines (Figure 1(b)). The shRNA construct strongly reduced the level of KIF3C protein in both cell lines (Figure 1(c)). In addition, the KIF3C ctDNA pCDH-CMV-MCS-EF1-Puro vector was transfected into U87 and U251 cell lines for the gain-of-function study. The empty vector was used as a negative control. Western blot analysis showed that KIF3C was increased in the transfection group compared to the empty vector group (Figure 1(c)).

### 3.2. Effects of KIF3C on the Migration and Invasion of Glioma Cells

**Figure 2:**
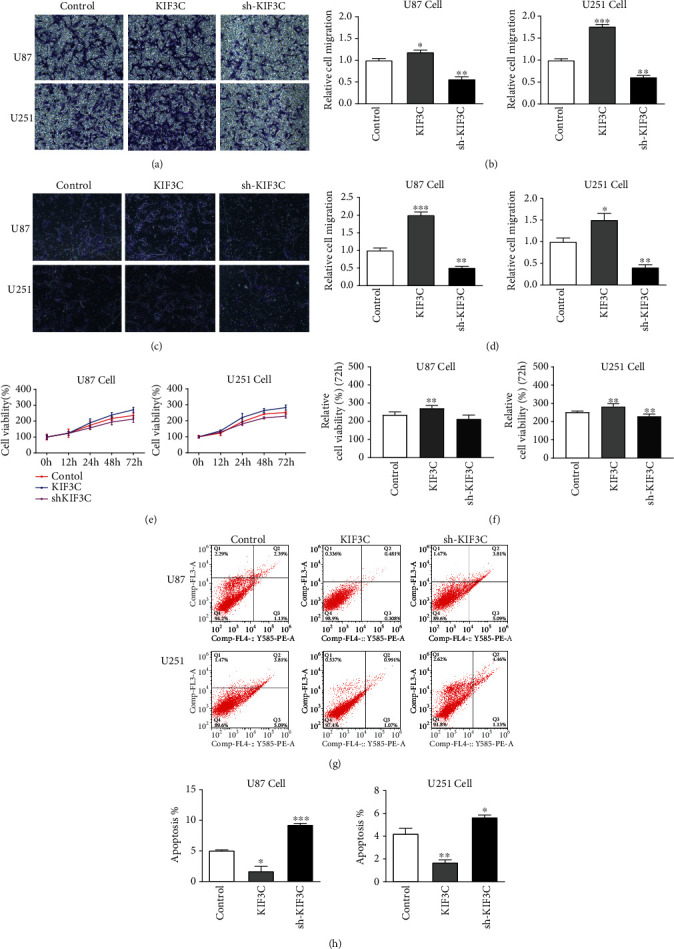
KIF3C promotes U87 and U251 cell migration, invasion, and proliferation and suppresses cell apoptosis *in vitro*. (a, b) Migration and (c, d) invasion of each cell line were evaluated by the transwell assay. The left panels show photos of representative fields (100x magnification), and the right panels show histograms of the results. (e, f) Proliferation was determined at the indicated time intervals using the Cell Counting Kit-8 reagent. (g, h) Cell apoptosis was measured using the annexin V-FITC/PI apoptosis detection kit. Data are expressed as the mean ± SD. Each experiment was repeated three times. ^∗^*P* < 0.05, ^∗∗^*P* < 0.01, and ^∗∗∗^*P* < 0.001.

As shown in Figures 2(a) and 2(b), cell migration in the KIF3C overexpression groups was significantly higher than that in the negative control groups, while the shRNA-KIF3C groups had the opposite effect on the two cell lines. Glioma cells had a higher invasive ability after increasing KIF3C expression, while the shRNA-KIF3C group had a less invasive ability in the two cell lines (Figures 2(c) and 2(d)). These results suggest that KIF3C can promote the invasive and migratory ability of U87 and U251 cells.

### 3.3. Effects of KIF3C on Proliferation and Apoptosis of Glioma Cells

The results showed that overexpression of KIF3C promoted U87 cell proliferation, while downregulation of KIF3C inhibited this effect compared to that of the control group (Figures 2(e) and 2(f)). Moreover, elevated KIF3C expression reduced U87 cell apoptosis, while suppressed KIF3C expression promoted U87 cell apoptosis (Figures 2(g) and 2(h)). KIF3C had the same effects on U251 cell lines (Figures 2(g) and 2(h)). These results suggest a potential role for KIF3C in promoting cell proliferation and suppressing cell apoptosis.

### 3.4. KIF3C Modulates the Expression of EMT and Apoptosis-Related Proteins

**Figure 3:**
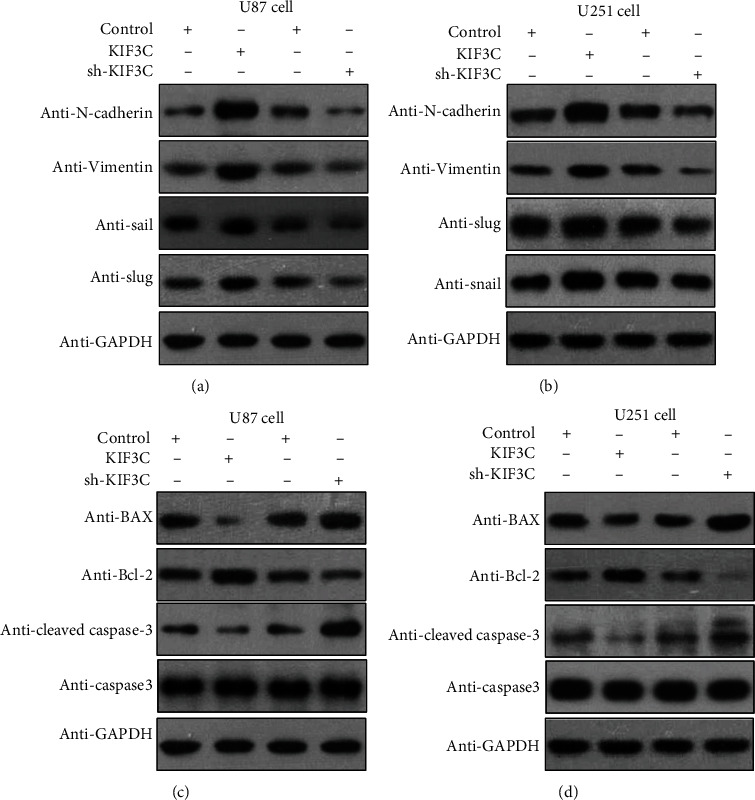
KIF3C modulates the expression of EMT and apoptosis-related proteins *in vitro*. (a) Overexpression of KIF3C increased N-cadherin, vimentin, snail, and slug expression to promote EMT *in vitro*, while KIF3C downregulation had the opposite effects. (b) Loss of KIF3C increased Bax levels and decreased Bcl-2 and cleaved caspase-3 expression, while overexpression of KIF3C had the opposite effects. (c, d) The same results were also found in U251 cells.

We found that in the GBM cell lines, the KIF3C upregulation group had increased expression levels of N-cadherin, vimentin, snail, and slug, inducing EMT, while KIF3C downregulation had the opposite effects (Figures 3(a) and 3(b)). Tumor cell growth relies on antiapoptotic mechanisms or activation of survival signals, such as the PI3K/AKT pathway. Bax has been demonstrated as an apoptosis promoter, while Bcl-2 and cleaved caspase-3 are apoptosis inhibitors. In our study, the KIF3C-downregulating cell group exhibited elevated levels of Bax and decreased levels of Bcl-2 and cleaved caspase-3 expression, while overexpression of KIF3C had the opposite effects on the two cell lines (Figures 3(c) and 3(d)).

### 3.5. KIF3C Regulates the PI3K/AKT Pathway in Glioma Cell Lines

**Figure 4:**
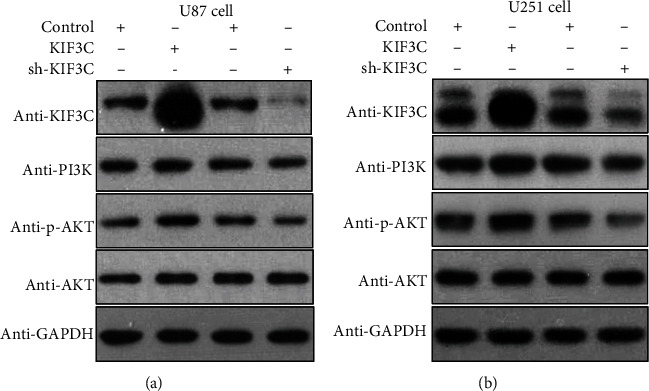
KIF3C was related to the PI3K/AKT pathway. (a, b) Western blotting demonstrated that overexpression of KIF3C increased PI3K and p-AKT level, while KIF3C downregulation had the opposite effects.

We further explored the mechanisms of KIF3C on proliferation, migration, and invasion in glioma cells. The reports indicated that the PI3K/AKT signaling pathway is activated to promote cell proliferation, migration, and invasion in GBM [23, 24]. Therefore, we investigated whether the PI3K/AKT pathway was affected by KIF3C in U87 and U251 cells. Western blotting results showed that in the glioma cell lines, the KIF3C downregulation group exhibited reduced levels of PI3K and phosphorylated AKT (Ser473). In addition, the KIF3C overexpression group had elevated levels of PI3K and phosphorylated AKT (Ser473), while the expression of total AKT protein did not change significantly (Figures 4(a) and 4(b)).

**Figure 5:**
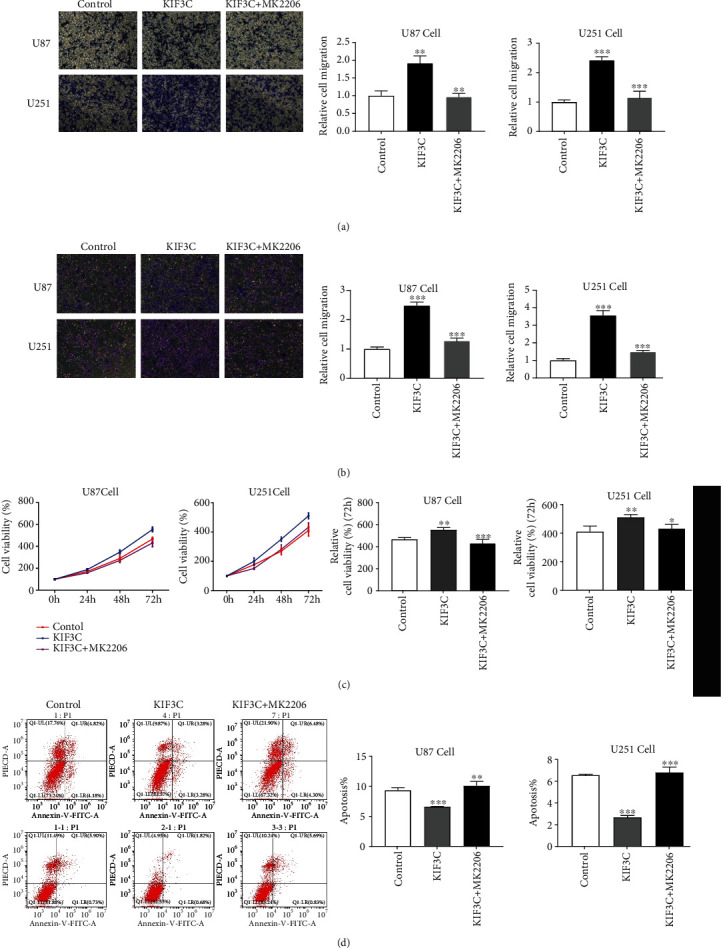
AKT inhibitor inhibits migration, invasion, and proliferation and enhances apoptosis of KIF3C-overexpressing cell lines. (a) Representative pictures of cell migration (left panel, magnification: 100x) and quantification of cell migration (right panel) (b) invasion, (c) proliferation, and (d) apoptosis *in vitro*. Data are expressed as the mean ± SD. Each experiment was repeated three times. ^∗^*P* < 0.05, ^∗∗^*P* < 0.01, and ^∗∗∗^*P* < 0.001.

**Figure 6:**
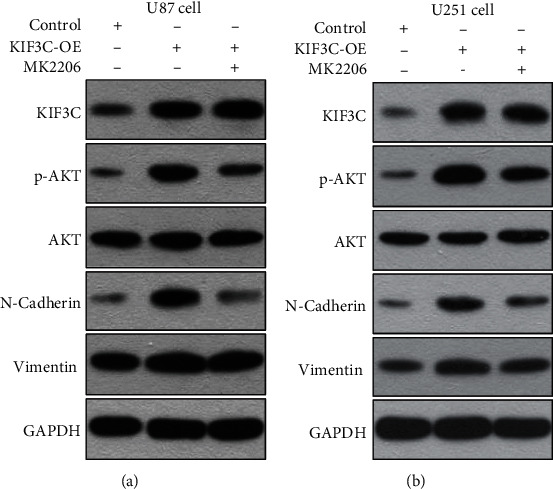
The AKT inhibitor MK2206 suppressed EMT in KIF3C-overexpressing cells. (a, b) Western blotting showed that N-cadherin was inhibited by the AKT inhibitor MK2206 in both KIF3C-overexpressing U87 and U251 cell lines.

We then treated U87 and U251 cells with the AKT inhibitor MK-2206 (Selleck Chemicals, USA) to investigate whether AKT phosphorylation could mediate KIF3C-induced glioma progression. We found that treatment of cells with MK-2206 reduced proliferation, migration, and invasion and promoted cell apoptosis (Figures 5(a)–5(d)). In addition, treatment of glioma cells with an AKT inhibitor suppressed the expression levels of N-cadherin and p-AKT in KIF3C-overexpressing cells (Figures 6(a) and 6(b)). Altogether, our data suggest that KIF3C is probably involved in the PI3K/AKT pathway and can induce EMT in glioma cell lines.

## 4. Discussion

Various studies have demonstrated the significant role of KIFs in cell division, intracellular transport, and cellular morphogenesis [5–8]. However, most of these studies focused on the structure and function of KIFs in normal cells. In recent years, several studies have identified the role of KIFs (KIF3A and KIF3B) in human cancers, confirming the effect of kinesin on the proliferation or invasion of tumor cells [14, 16, 21]. Since KIF3C is expressed in the CNS, we investigate whether KIF3C has a potential role in glioma.

In this study, we conducted comprehensive research *in vitro* to explore the function of KIF3C in glioma. We first found that overexpression of KIF3C promoted cell proliferation, migration, and invasion and suppressed cell apoptosis, while silencing of KIF3C had the opposite effects on both glioma cell lines. Our results were consistent with those of a previous study in breast cancer cells, indicating the potential clinical value of KIF3C in glioma [21].

Previous evidence has suggested that KIF members influence the function of different human tumor cells through various signaling pathways. KIF3A promotes cell proliferation and invasion in advanced prostate cancer via the *Wnt* signaling pathway [14]. The downregulation of KIF3C expression inhibits tumor growth and metastasis in breast cancer by inhibiting TGF-*β* signaling [21]. It has been reported that the PI3K/AKT pathway regulates tumor cell survival, growth, motility, angiogenesis, and metabolism in a variety of cancers, including GBM [25, 26]. Inhibition of the PI3K/AKT pathway may result in GBM cell death and slow tumor progression [27, 28]. It is reported that KIF3C was related to the PI3K/AKT pathway, which lacked a confirmatory experiment [29]. Therefore, we investigated whether KIF3C was involved in the PI3K/AKT pathway in glioma cells. In this study, western blotting results showed that overexpression of KIF3C elevated the levels of PI3K and p-AKT, while silencing of KIF3C reduced the levels of PI3K and p-AKT. In addition, EMT is identified as a driver of invasion and metastasis in different types of epithelial cancers [30]. Our data supported that overexpression of KIF3C could increase the expression of N-cadherin, vimentin, snail, and slug to promote EMT *in vitro*, while silencing of KIF3C had the opposite effects. Furthermore, we found that treatment of U87 and U251 cells with the AKT inhibitor MK-2206 reduced proliferation, migration, and invasion and promoted cell apoptosis *in vitro*. Treatment of glioma cells with an AKT inhibitor suppressed the expression levels of N-cadherin and p-AKT in KIF3C-overexpressing cells. Taken together, these results indicate that KIF3C may be involved in the PI3K/AKT pathway and induce EMT in glioma cells.

Studying the distribution of KIF3C may provide important clues as to the possible functions of this molecule. KIF3C is overexpressed in breast cancer tissues, and such high KIF3C expression is also associated with tumor recurrence and lymph node metastasis [21]. However, in the CNS, the KIF3C peptide can efficiently induce glioma-reactive cytotoxic T lymphocytes (CTLs) from patients, indicating an effective peptide-based immunotherapy for glioma patients [31]. Of course, the role of this protein should be further investigated before clinical use. The high expression of KIF3C in the nervous system during ontogenesis and its upregulation during neuronal differentiation have been observed and identified [20]. As for the lack of relevant research, we hypothesize that the expression level of KIF3C in different-grade gliomas (grades I to IV) may vary because of differentiation and dedifferentiation. Further study will explore the expression and function of KIF3C in high- and low-grade glioma tissues and *in vivo*.

## 5. Conclusion

We first confirm that overexpression of KIF3C promotes proliferation, migration, and invasion and inhibits apoptosis in glioma cells. Regarding the mechanism by which KIF3C regulates these functions, our results provide clues that the PI3K/AKT pathway and EMT may be involved, which require further investigation. These findings suggest that KIF3C might serve as a potential biomarker for further basic research or clinical management of glioma.

## Figures and Tables

**Table 1:** Primary antibodies used in this study.

Primary source	Antibody clone	Company	Code	Dilution
KIF3C	Rabbit	Proteintech	14333-1-AP	1 : 1000
PI3K	Rabbit	CST	4257	1 : 1000
AKT	Rabbit	CST	4691	1 : 1000
p-AKT	Rabbit	CST	4060	1 : 1000
Vimentin	Rabbit	CST	5741P	1 : 1000
Slug	Rabbit	CST	4585P	1 : 1000
Snail	Rabbit	CST	38879P	1 : 1000
BCL-2A1	Rabbit	CST	35596/4926	1 : 1000
BAX	Rabbit	Proteintech	50599-2-lg	1 : 1000
Cleaved caspase-3	Rabbit	CST	9664T	1 : 1000
Caspase-3	Rabbit	Abcam	ab13585	1 : 1000
GAPDH	Rabbit	Proteintech	10494	1 : 10000
N-cadherin	Rabbit	Proteintech	22018-1-AP	1 : 1000

Abbreviation: KIFs: kinesin superfamily proteins; PI3K: phosphatidylinositol 3-kinase; AKT: protein kinase; GAPDH: glyceraldehyde-3-phosphate dehydrogenase; CST: Cell Signaling Technology.

**Table 2:** Secondary antibodies used in this study.

Secondary antibody	Dilution	Company
Anti-mouse	1 : 3000-1 : 5000	Proteintech
Anti-rabbit	1 : 3000-1 : 5000	Proteintech

## Data Availability

The datasets generated and/or analyzed during the present study are available from the corresponding author upon reasonable request.
